# Post-Exercise Nutrition Knowledge and Adherence to Recommendations Among Amateur Endurance Athletes

**DOI:** 10.3390/nu17223629

**Published:** 2025-11-20

**Authors:** Lilla Csanaky, Ágnes Czeglédiné Asztalos, Dorottya Tóth, Éva Polyák, Mária Figler

**Affiliations:** 1 Doctoral School of Health Sciences, Faculty of Health Sciences, University of Pécs, 7621 Pécs, Hungary; agnes.asztalos@etk.pte.hu (Á.C.A.); dorottya.toth@etk.pte.hu (D.T.); 2 Institute of Nutritional Sciences and Dietetics, Faculty of Health Sciences, University of Pécs, 7621 Pécs, Hungary; eva.polyak@etk.pte.hu (É.P.); maria.figler@pte.hu (M.F.); 3 Institute of Physiotherapy and Sport Science, Faculty of Health Sciences, University of Pécs, 7621 Pécs, Hungary; 4 2nd Department of Internal Medicine and Nephrology Center, University of Pécs, 7624 Pécs, Hungary

**Keywords:** endurance athletes, post-exercise nutrition, nutrition knowledge, recovery, PENRAS, triathlon

## Abstract

**Background/Objectives**: Optimal post-exercise nutrition is critical for maximizing recovery and subsequent performance. However, athletes often lack knowledge of guidelines, leading to suboptimal practices, particularly inadequate carbohydrate intake for glycogen resynthesis. This study aimed to assess the adherence of Hungarian endurance athletes to nutritional recommendations, identifying deficits and guiding the development of effective educational strategies. **Methods**: A cross-sectional study surveyed 113 amateur Hungarian endurance athletes (mean age 40.04 ± 9.89 years) training ≥ 3 times/week using a self-developed online questionnaire. A ten-item composite measure, the Post-Exercise Nutrition Recommendation Adherence Score (PENRAS, max 10 points), was calculated to assess adherence. Statistical analyses, including ANOVA and regression, were used to explore factors influencing PENRAS and nutritional practices. **Results**: The overall mean PENRAS was 5.32 ± 1.52, indicating room for improvement. The most pronounced deficit was observed in quantitative knowledge, with only 1.8% of participants correctly identifying the optimal carbohydrate content required for rapid glycogen resynthesis. Concurrently, high protein content (58.4%) was mentioned by a higher percentage than high carbohydrate content (52.2%) as an aspect of post-exercise meal planning. Triathletes had significantly higher PENRAS than runners (6.28 vs. 4.97, *p* = 0.001). Higher PENRAS was also significantly associated with consultation with a dietitian (*p* = 0.018). Reliance on professionals positively predicted knowledge, while online sources were a significant negative predictor. Higher PENRAS was associated with better meal planning and earlier post-exercise meal timing. **Conclusions**: Endurance athletes’ post-exercise nutritional practices are suboptimal. The findings emphasize the need for targeted interventions prioritizing education on carbohydrate intake and redirecting athletes towards evidence-based information to improve adherence and performance outcomes.

## 1. Introduction

The close relationship between athletic performance and nutrition has become increasingly evident over the past few decades. Consequently, to optimize performance and maximize training adaptations, it is crucial for athletes to ensure adequate nutritional intake and establish dietary strategies that are precisely customized to their training load. By appropriately scheduling their macronutrient intake, athletes can support their training goals, whether they aim to optimize body composition, rapidly replenish glycogen stores, or promote protein synthesis [1].

According to study results, athletes’ nutrition is often not aligned with their training load and fails to provide the necessary nutrient supply for high-level performance [2,3]. Achieving appropriate performance requires a sport-specific approach. In the case of endurance sports, adequate carbohydrate intake is particularly important. However, in today’s diet culture, which often stigmatizes carbohydrates and is permeated by misconceptions, both amateur and professional athletes frequently fail to reach the necessary carbohydrate intakes tailored to their training load [4,5,6].

The root of inadequate nutrient intake is often a lack of knowledge regarding scientific guidelines [7,8,9]. Athletes typically base their habits regarding sports nutrition and the use of dietary supplements on their own experiences, the accounts of other athletes, or information from online sources, with few being familiar with professional recommendations [7,8].

However, the effective translation and application of post-exercise nutritional recommendations often remain suboptimal [7]. When considering the composition of post-exercise meals, athletes often encounter recommendations tailored for resistance-type training. Applying these recommendations in the context of endurance sports may not optimally support the achievement of maximum athletic performance. For example, many athletes consume protein-containing dietary supplements in the hope of faster recovery and a more favorable body composition (i.e., greater protein synthesis and increased fatty acid oxidation), but they often overlook replenishing their depleted glycogen stores. According to dietary records, a higher proportion of amateur runners meet the recommended minimum protein intake after exercise than they do for carbohydrate intake [8].

Key determinants of carbohydrate utilization during exercise include the volume (total duration) and intensity of the activity, as well as the athlete’s total energy expenditure, and exercise economy, the latter being important since the energy cost required for a given performance output is highly individual. Higher-intensity exercise leads to a greater rate of carbohydrate oxidation, making muscle glycogen and blood glucose the predominant energy sources. In contrast, at lower intensities, a larger proportion of energy is derived from fat oxidation, thereby preserving glycogen stores.

The rate and proportion of carbohydrate utilization vary substantially across different sports disciplines, depending on the nature of the exercise and its metabolic demands. In endurance-based events such as marathon running, road cycling, or cross-country skiing, athletes perform sustained, moderate-to-high-intensity exercise that places a high demand on carbohydrate availability. The primary nutritional goal in these cases is to maintain a high rate of exogenous carbohydrate oxidation, which underlies the widely recommended intake of 60–90 g⋅h^−1^ to spare muscle glycogen [10]. Conversely, ultra-endurance events, such as Ironman triathlons or ultramarathons, involve a lower relative intensity but extremely high total volume and cumulative energy expenditure. Although the reliance on fat oxidation is higher, the sheer duration of these events can necessitate carbohydrate intakes of up to 120 g⋅h^−1^ (when using multiple transportable carbohydrates) to mitigate severe energy deficits and sustain central nervous system function [10].

During long-duration, high-intensity training sessions, muscle and liver glycogen stores become depleted, a process that contributes to fatigue and diminishes performance [10]. According to current sports nutrition guidelines, following an endurance-type training load, an intake of 1–1.2 g·BW^−1^⋅h^−1^ of carbohydrate is recommended during the four-hour period after exercise to ensure the fastest possible replenishment of glycogen stores in the muscles and liver [11,12,13,14,15,16]. The importance of rapid replenishment is particularly high when the recovery window is narrow (i.e., less than 8 h between training sessions), a highly relevant factor for triathletes and other endurance athletes due to their high training volume. The rate of glycogen resynthesis is highest during the first 1–2 h post-exercise. Consuming adequate carbohydrates in this critical period is essential for athletes engaging in frequent and intensive endurance training, as it enables proper recovery before the next training session [13,14,17].

Questionnaires designed to assess nutritional knowledge are often general and, in some cases, time-consuming to complete [18,19,20,21,22,23,24,25,26]. Even if they include a few questions regarding recovery, this is often insufficient to fully assess the complexity of the subject. The importance of this topic is underscored by a survey utilizing the Comprehensive Evaluation of Athlete’s Knowledge Questionnaire (CEAC-Q), specifically designed to assess carbohydrate knowledge among endurance athletes. Within this survey, the lowest rate of correct answers was recorded in the questionnaire’s recovery section [27]. In the case of the PEAKS-NQ, selecting appropriate recovery meals/nutrients is similarly noted as one of the more challenging sports nutrition concepts [23]. Ultra-endurance athletes, on the other hand, reached significantly higher scores in the recovery questions and performed significantly worse in the fluid and supplement questions [28]. To address this identified gap, we developed the Post-Exercise Nutrition Recommendation Adherence Score (PENRAS) as a specific, multi-faceted composite measure. Our research aims to comprehensively assess Hungarian endurance athletes’ knowledge of and adherence to post-exercise nutritional recommendations—a key area not extensively covered in the existing literature. Furthermore, we investigated the influence of sociodemographic factors, sport discipline, and sources of nutrition information on both knowledge and practical habits. Obtaining this insight is crucial for developing more effective educational strategies for this population, as the pivotal role of sports nutrition professionals in significantly improving athletes’ nutritional knowledge and practices is well established [29,30,31].

## 2. Materials and Methods

### 2.1. Study Design

The present study investigated the knowledge and habits related to post-workout nutrition among a cohort of Hungarian endurance athletes, using a self-developed online questionnaire between March 2022 and June 2023. The questionnaire (Supplementary Materials) was administered via Google Forms software (Google LLC, Mountain View, CA, USA). A link to the questionnaire was delivered to the participants via email and various social media platforms. The questionnaire was actively available and collected responses throughout this period, from March 2022 until it was closed in June 2023.

Respondents were recruited in part through expert sampling, given that the authors had connections with endurance athlete teams. Additionally, snowball method was applied, as the study was promoted online through a variety of social media platforms. Individuals were eligible to take part in this study if they were healthy endurance athletes aged over 18 years and engaged in endurance training at least three times per week. We specified in the recruitment advertisement that we were seeking responses exclusively from amateur endurance athletes. This was intended to exclude professional or elite athletes whose training loads and access to nutritional resources may differ significantly from the general amateur athletic population. The research population consisted of non-elite, dedicated amateur triathletes and runners who participate in races recreationally.

All experimental procedures were approved by the local Ethics Committee (Scientific and Research Committee of the Medical Research Council, Hungary; Decision IV/573-1/2022/EKU) and were conducted in accordance with the Declaration of Helsinki. All participants were provided with information about the aim of the research and were informed of the confidential use of their data for statistical purposes, and that the results would be published in scientific presentations and academic papers. Participants provided their electronic informed consent before completing the questionnaire.

**Figure 1:**
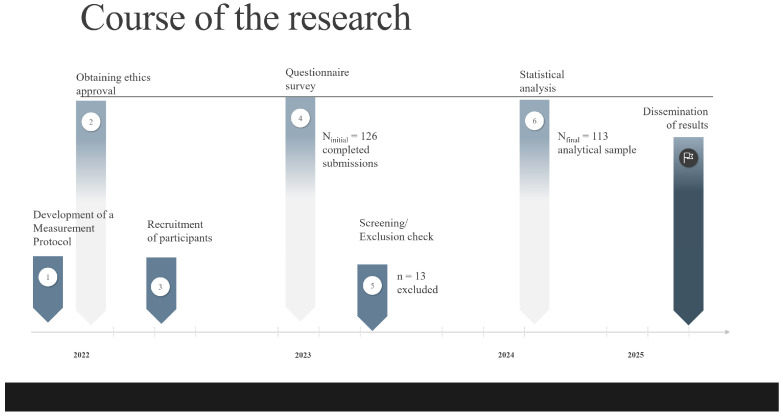
Course of the Study and Participant Flow.

The overall course of the research, including the timeline for data collection and participant flow, is summarized in Figure 1.

### 2.2. Participants

A total of 126 completed questionnaires were initially received. Thirteen (13) participants were excluded from the final analysis because their self-reported training frequency did not meet the minimum three-times-per-week inclusion criterion. The final analytical sample consisted of 113 participants with a mean age of 40.04 years (*SD* = 9.89), ranging from 19 to 70 years. The age distribution of respondents was close to symmetrical, with a median age of 41 years. The gender distribution in our sample was relatively balanced, with 57.5% (*n* = 65) female and 42.5% (*n* = 48) male respondents. The majority of respondents were highly educated, with 78.8% (*n* = 89) possessing a university degree. The remaining 21.2% had completed secondary education, including vocational school (*n* = 11) and high school (*n* = 13).

In accordance with the inclusion criteria, the sample consisted of endurance athletes performing endurance training a minimum of three times per week. 23.9% (*n* = 27) of the participants identified themselves as triathletes. 43.4% of the sample exclusively participated in running, while one participant reported cycling as the sole endurance activity. 17.7% practiced both running and cycling on a regular basis, 6.2% combined running and swimming, and 8% regularly performed running, cycling, and swimming without participating in triathlon competitions. For later statistical analysis, participants were classified into three groups based on their sports: runners (those who exclusively ran), triathletes, and mixed endurance athletes (all other participants engaging in multiple endurance sports). This categorization was based solely on the discipline(s) practiced by the athletes and not on self-reported weekly training volume. Group comparisons of demographic variables are presented in Section 3.

### 2.3. Questionnaire

The questionnaire was created in Hungarian and consisted of multiple sections. The first section included demographic questions (gender, age, and highest educational level) and basic anthropometric data (height and weight). The second section focused on general dietary habits, including the presence of food allergies and intolerances, dietary patterns followed, nutrition-related goals, the number of meals consumed on recovery and training days, meal planning routines, nutrition periodization strategies, sources of nutrition information, and prior consultation with a dietitian or sports nutritionist. Since no validated questionnaire specifically addressing post-exercise nutrition knowledge and habits of endurance athletes was available at the time of the survey, we developed our own questions to assess this crucial topic. Given the study’s focus on this area, multiple questions were dedicated to key aspects, including the timing and composition of post-exercise meals, knowledge about optimal recovery windows, as well as awareness of the optimal carbohydrate content of post-workout meals. Participants were asked about their typically consumed foods and beverages after exercise, as well as factors considered when choosing their post-exercise meal. Further sections included several questions related to the supplements used by the athletes and their fluid intake, as well as their training habits. All questions in the online questionnaire were set as mandatory response fields. This method effectively eliminated item-level missing data within the accepted *n* = 113 sample. Consequently, the statistical analysis did not require imputation or handling of internal missing values for the core variables.

### 2.4. Data Analysis

For the purpose of this study, the collected data were systematically processed and analyzed in four distinct stages: analysis of demographic data, the development of the Post-Exercise Nutrition Recommendation Adherence Score (PENRAS), mapping paths leading to higher PENRAS, and investigating the associations between PENRAS and dietary habits.

The initial stage of analysis focused on the compilation and processing of demographic and foundational sports-related data for the respondents. Descriptive statistics were used to interpret demographic statistics and response frequencies. For the presentation of continuous variables, means and standard deviations (*SD*) were used, while categorical variables were presented as frequencies and percentages.

For later analysis, participants were grouped into the following categories: runners, triathletes, and mixed endurance athletes. Respondents who participated in at least two endurance sports were classified as mixed endurance athletes. One respondent identified exclusively as a cyclist and could not be assigned to the above categories, so his data were excluded from analyses broken down by sports groups. Demographic differences between sport groups were analyzed via Pearson’s chi-square tests and Kruskal–Wallis tests.

In addition to the demographic description, frequency analysis was used to examine the dietary patterns and nutrition-related goals of respondents. To evaluate the potential differences between these nutrition-related goals, a series of McNemar’s tests was performed.

In the second block, a composite score was created that we named PENRAS (Post-Exercise Nutrition Recommendation Adherence Score). We assessed the athletes’ nutritional knowledge and level of adherence to guidelines related to recovery nutrition in the context of endurance training. This was done using a set of self-developed questions. To create a composite score, 10 individual response options were selected from these questions. This part of the questionnaire included two types of questions: those measuring theoretical knowledge and those assessing actual practices. For the knowledge-based questions, response options included the correct quantities and timeframes, along with smaller and larger values to evaluate accuracy. Other items specifically investigated how athletes apply these habits in practice. The combination of knowledge-based and practice-based items was conceptually driven by the aim to measure overall compliance to post-exercise nutrition recommendations. Effective adherence requires both the necessary theoretical foundation (knowledge) and the successful application of this knowledge in a real-world scenario (practice). Each of the ten items was analyzed individually using frequency data.

To assess which areas require the most focus in educational activities, we established three reference ranges based on the percentage of correct answers for each item: “low” (0–39%), “moderate” (40–69%), and “high” (70–100%). This categorization was based on the criteria thresholds used in a study assessing knowledge of carbohydrate guidelines of endurance athletes [27].

The ten items used as indicators to assess the participants’ post-exercise nutrition knowledge and habits were as follows: the optimal timing of post-exercise meals; the key aspects considered when selecting post-exercise nutrition; the recommended carbohydrate content of a post-exercise meal; and whether participants intentionally increase their carbohydrate and protein intake on days involving more intense training sessions.

The scoring procedure for each item was defined according to specific criteria. For multidimensional response items, participants could provide multiple correct elements. For example, in the assessment of key aspects of post-exercise nutrition selection, one point was assigned for mentioning each of the following: high carbohydrate, high sodium, high protein, high energy content, easy digestibility, and low fat content. A summative approach was used, awarding one point for each correctly identified component, allowing a nuanced assessment of partial knowledge. For quantitative items (e.g., recommended carbohydrate intake), the scoring procedure was designed to assign partial credit for responses that demonstrated a degree of correct knowledge, such as awarding 0.5 points for the two answers closest to the optimum, while only the fully correct response received the full point.

According to current guidelines, the optimal timing for nutrient intake to facilitate post-exercise glycogen synthesis and rapid recovery is considered to be within two hours. Consequently, any timing options presented within this two-hour window (within 30 min/60 min/2 h) were deemed correct [13,14,17]. Regarding the optimal carbohydrate content of the post-exercise meal, one point was given to the correct answer (1–1.2 g·BW^−1^⋅h^−1^), and 0.5 points were given to the two options closest to the correct answer (0.8–1 g/BW/h and 1.2–1.6 g·BW^−1^⋅h^−1^). This methodology was based on the guidelines published by the International Society of Sports Nutrition and the Position of the Academy of Nutrition and Dietetics, Dietitians of Canada, and the American College of Sports Medicine: Nutrition and Athletic Performance [12].

The minimum score that could be obtained was 0, and the maximum reachable score was 10. Descriptive statistics, including the mean, standard deviation, distribution characteristics (median, minimum and maximum values), and frequencies with their corresponding percentages, were reported for this scale. The PENRAS demonstrated an acceptable level of normality (skewness = −0.30, kurtosis = −0.50).

The third analytical block focused on evaluating the predictors of PENRAS, specifically examining the influence of demographic and sport-related factors and analyzing the role and predictive value of various information sources utilized by the participants. To evaluate the association of demographic and sport-related factors on the PENRAS, univariate ANOVA was conducted, with sport group, gender, age, education level, and previous consultation with a dietitian entered as independent variables. Weekly endurance training volume was included in the model as a separate covariate to statistically control for its potential effect on the PENRASs. Due to the limited sample size, only main effects were tested, and no interaction terms were included. Partial eta squared (*η*^2^) values were reported as effect sizes for significant predictors.

Additionally, we analyzed the influence of various information sources on the participants’ knowledge score. The extent to which respondents utilized specific sources of information (e.g., coaches, online platforms, nutritional professionals) was measured on a 1–5 Likert scale. Differences in the ratings of these scores were then assessed using a Wilcoxon signed-rank test. To analyze the predictive value of the information sources on PENRASs, a linear regression analysis was performed. The assumptions of the linear regression were analyzed and met.

The fourth analytical block aimed to characterize the participants’ dietary and meal planning habits using descriptive statistics, and subsequently employed ordinal regression analysis to investigate the association of various factors, including the PENRAS, on these nutritional practices. Descriptive statistics were used to characterize the general dietary habits of the sample. Frequencies and percentages were calculated to analyze the macronutrient periodization according to training load and meal planning habits of the athletes.

Ordinal regression analyses were applied (PLUM models) to explore the relationship of various predictors (e.g., PENRAS, gender, age, sport group, consultation with a dietitian, training volume) on meal planning habits, timing of post-exercise meals, timing of energy-containing beverage consumption, and the frequency of consumption of specific food items in the post-exercise period. Model fit was evaluated using chi-square statistics, and explanatory power was interpreted based on Nagelkerke *R*^2^ and McFadden *R*^2^ values.

All data analyses were conducted using IBM SPSS Statistics software (version 28.0.0.0, IBM Corp., Armonk, NY, USA). The significance level was set at *p* < 0.05 for all statistical tests.

## 3. Results

### 3.1. Comparison of Demographic Characteristics Between Sport Groups

Before the main analyses, demographic characteristics of the three sport groups (runners, mixed endurance athletes, and triathletes) were compared. A detailed summary of these characteristics is presented in Table 1. According to Pearson’s chi-square test, there was no statistically significant difference between the three groups as regards the gender distribution of the participants (*p* = 0.302). The proportion of female athletes was 65.3% among runners, 50.0% in the mixed sports group, and 51.9% among triathletes. Similarly, the ratio of participants who had previously consulted a dietitian did not show a statistically significant difference between groups (*p* = 0.457). An observable trend suggested that athletes engaging in several or more complex endurance sports were more likely to consult a dietitian. However, this correlation did not reach the level of statistical significance, possibly due to the limited sample size and low statistical power. The proportions of athletes reporting a consultation with a nutrition professional before joining our study were as follows: 49.0% of runners, 58.3% of mixed endurance athletes, and 63.0% of triathletes. Age and educational background of the participants in the three sports groups were compared using Kruskal–Wallis tests. No statistically significant difference between the groups was observed in terms of age (*p* = 0.141) or level of education (*p* = 0.500).

### 3.2. Diets and Goals Related to Nutrition

Of the 113 people who took part in the survey, 40.7% did not follow any specific diet. Based on self-declaration, 7.1% of participants followed a low-carb diet, 3.5% a low-fat diet, 10.6% a gluten-free diet, and 10.6% excluded dairy. 7.1% considered themselves vegetarian, 10.6% vegan, 3.5% practiced intermittent fasting (time-restricted eating), and 2.7% described their diet as flexitarian.

The analysis of nutrition-related goals revealed that most respondents (*n* = 77; 68.1%) primarily focused on improving their sports performance. This goal was indicated by a significantly higher proportion of participants compared to body image-related goals, such as weight loss (*n* = 29; 25.7%), getting lean (*n* = 25; 22.1%), and gaining muscle mass (*n* = 24; 21.2%) (all *p* < 0.001). Based on McNemar tests, no statistically significant differences were found in the frequency of these goals (e.g., weight loss vs. muscle gain: *p* = 0.486). 23.0% of respondents (*n* = 26) reported not having a specific nutritional goal. The least frequently mentioned nutrition-related goal was to alleviate gastrointestinal complaints, reported by 12.4% (*n* = 14). When compared to weight loss, a significant difference was found (*p* = 0.018), while marginal significance was observed when compared to muscle gain (*p* = 0.099), getting lean (*p* = 0.082), and having no specific nutrition-related goal (*p* = 0.082).

### 3.3. Post-Exercise Nutrition Recommendation Adherence Score (PENRAS)

Ten answers were taken into consideration when evaluating the extent to which participants’ post-exercise and periodized nutrition practices align with evidence-based sport nutrition guidelines.

Regarding the optimal timing of the first post-exercise meal, 84.1% of respondents were aware of the importance of energy intake within 2 h for optimal recovery. It is worth highlighting that 40.7% of participants indicated an even stricter time frame, selecting within 30 min post-workout.

When asked about the optimal carbohydrate content of meals consumed in the hours following endurance exercise, more than half of the participants reported being unaware of the current recommendations. Among those athletes who attempted to select the correct answer, a significant proportion indicated either substantially lower or higher amounts of carbohydrates.

The overall PENRAS is weighted predominantly toward self-reported practice (adherence), with only two items—the optimal timing and the optimal carbohydrate content —measuring factual knowledge. A significant disparity exists between these two knowledge items: While the majority of athletes demonstrated high awareness regarding optimal timing, the precise quantitative recommendation for carbohydrate intake was correctly identified by only a low proportion of participants.

**Figure 2:**
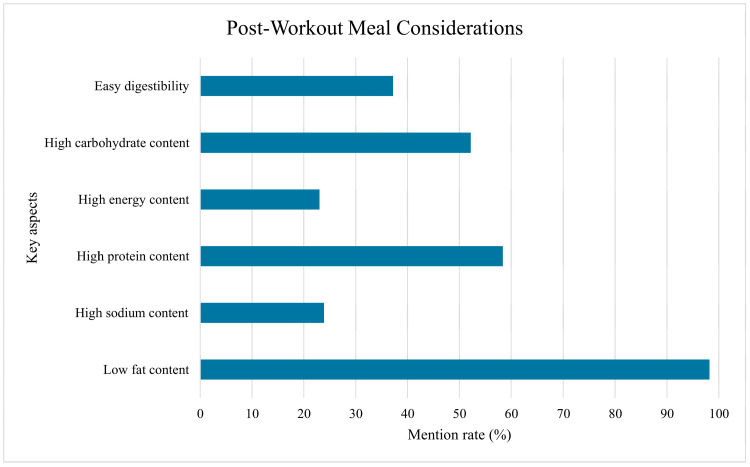
Post-workout meal considerations of participants.

The athletes’ considerations regarding the desired composition of their post-workout meal are summarized in Figure 2. When planning their post-workout meal, both high protein (58.4%) and high carbohydrate (52.2%) content were considered important. Notably, high protein content was mentioned at a higher rate than high carbohydrate content. Easy digestibility was marked as a relevant aspect by only 37.2% of athletes. High energy and high sodium content of the post-exercise meal were considered important by a similar proportion of respondents: 23.0% and 23.9%, respectively. Almost all participants (98.2%) were aware that high fat content is not beneficial in post-workout meals.

On more intense training days, 85.0% of respondents increase their carbohydrate consumption, while 55.8% indicated consuming more protein when their training load is higher than usual. The distribution of answers to the ten questions included in the PENRAS-indicator is presented in Table 2.

**Figure 3:**
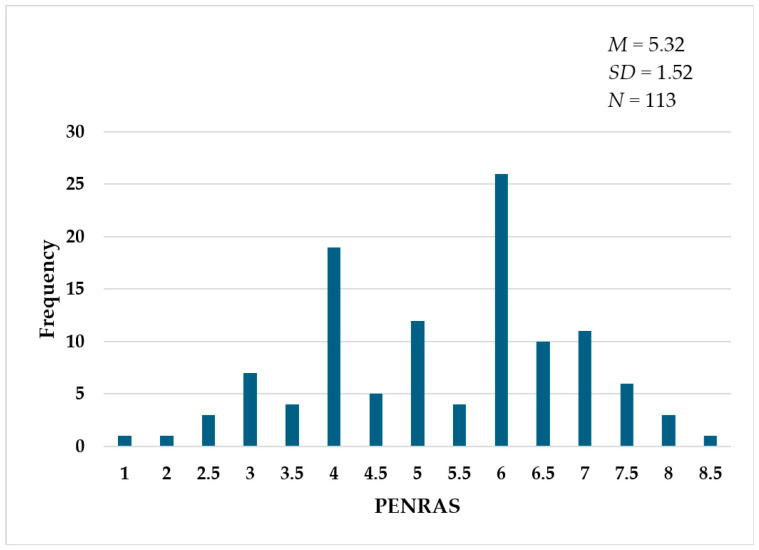
Distribution of PENRAS results among study participants.

The maximum achievable score was 10 points. 64.6% of the participants reached at least half of the maximum, so at least 5 points. The overall average score of respondents was 5.32 (*SD* = 1.52), with a median score of 6. The highest score recorded was 8.5 points, achieved by a single participant. The distribution of scores across the study sample is illustrated in Figure 3.

#### 3.3.1. Sport-Demographic Factors Influencing PENRAS

For the analysis of the effects of demographic and behavioral factors on the PENRAS, ANOVA was conducted with the following predictors: age, gender, sports group, training volume, education, and prior consultation with a dietitian. The overall model was statistically significant, *F*(7, 104) = 4.39; *p* < 0.001; with an *R*^2^ of 0.228 (adjusted *R*^2^ = 0.176). According to the result of our analysis, a significant main effect of sport group was found (*F*(2, 104) = 5.91; *p* = 0.004; part. *η*^2^ = 0.10). Post hoc comparisons using the Tukey HSD test indicated that triathletes had a significantly higher level of knowledge about post-training nutrition than runners (*p* = 0.001) and the mixed-endurance group (*p* = 0.007). The mean scores for the triathletes, runners, and the mixed-endurance athletes were 6.28 (*SD* = 1.30), 4.97 (*SD* = 1.46), and 5.14 (*SD* = 1.50), respectively. No significant difference was found between the runner and mixed groups (*p* = 0.854). When controlled for the effect of the sport groups, training volume did not have an additional significant effect on athletes’ PENRASs (*F*(1, 104) = 0.11; *p* = 0.742; part. *η*^2^ < 0.01). Beside the sport group, age was a predictor that showed a statistically significant effect, (*F*(1, 104) = 6.75; *p* = 0.011; partial *η*^2^ = 0.06) on PENRAS; older participants tended to reach slightly lower PENRASs. In contrast, gender (*F* (1, 104) = 0.11; *p* = 0.745; partial *η*^2^ < 0.01) and education (*F*(1, 104) = 0.01; *p* = 0.917; partial *η*^2^ < 0.01) did not have significant effects on PENRAS in our sample. Previous consultation with a dietitian was also a significant predictor for PENRAS in our sample (*F*(1, 104) = 5.73; *p* = 0.018; partial *η*^2^ = 0.05). The mean score of respondents who consulted a dietitian, was 5.65 (*SD* = 1.44), while athletes without experience with a dietitian reached an average score of 4.91 (*SD* = 1.54).

#### 3.3.2. Information Sources and Their Impact on PENRAS

**Figure 4:**
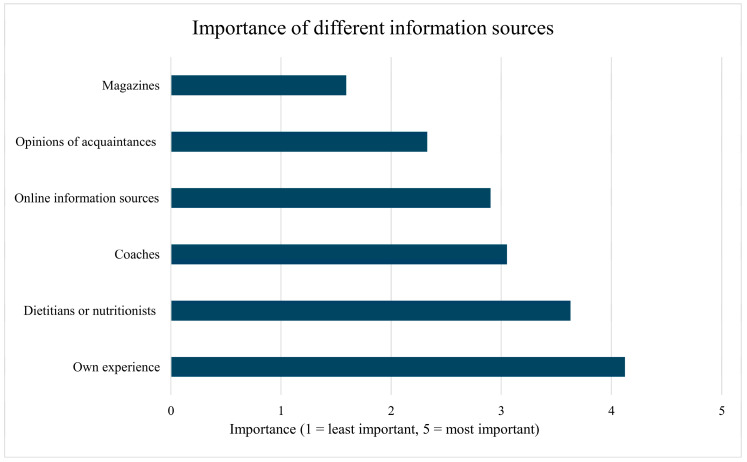
Importance of information sources among study participants.

Respondents were asked to rate the importance of different sources they rely on when it comes to shaping their dietary habits and extending their knowledge (1 = least important, 5 = most important source of information). The importance of different information sources is illustrated in Figure 4. Differences in the significance of the various information sources were analyzed using Wilcoxon signed-rank tests. According to our results, respondents mostly relied on their own experiences (*M* = 4.12; *SD* = 1.00), which proved to be significantly more important than the second most mentioned source (*p* = 0.005), dietitians or nutritionists (*M* = 3.63; *SD* = 1.53). These professionals seemed to be significantly (*p* < 0.001) more important information sources than coaches (*M* = 3.05; *SD* = 1.49). Although reliance on coaches for nutritional information was moderate, it was higher than the importance of online information sources; however, this difference did not reach the level of significance (*p* = 0.309; *M* = 2.90; *SD* = 1.20). The opinions of acquaintances were reported to be significantly less influential (*p* < 0.001; *M* = 2.33; *SD* = 1.08) but still more important than magazines (*M* = 1.59; *SD* = 0.85). A significant difference (*p* < 0.001) was observed between the importance of these factors as well. Overall, participants placed the greatest emphasis on their own experience and professional guidance in their decision-making related to dietary habits.

We also examined whether there is a relationship between the information sources used by the respondents and their level of knowledge. To predict the PENRAS, linear regression analysis was applied. The regression model was significant (*F*(6, 106) = 6.06; *p* < 0.001) and explained 25.5% of the variance in knowledge level. Out of the predictor variables, three were found to be statistically significant: reliance on coaches (*β* = 0.27; *p* = 0.011) and nutritional professionals (*β* = 0.21; *p* = 0.045) had a significant positive effect on nutrition knowledge, while seeking nutritional information from online sources emerged as a significant negative predictor (*β* = −0.26; *p* = 0.006). The reliance on other information sources (own experience, acquaintances, and magazines did not have a significant effect on PENRAS in our sample (*p* > 0.05).

### 3.4. Associations Between PENRAS and Sports Nutrition Behavior

#### 3.4.1. Daily Dietary Routine

Our sample of endurance athletes seems to periodize their nutrition to their training load: on days with higher training intensity, 63.7% of respondents reported consuming larger quantities of food. 24.8% eat less on days with a higher training load, and 11.5% do not alter their food intake based on exercise intensity. Regarding macronutrients, respondents primarily focus on the periodization of their carbohydrate intake: 85.0% reported consuming more carbohydrates on days with a higher training load, 55.8% increased their protein intake, and only 30.1% increased their fat intake on these days.

Athletes in our sample tend to plan their meals spontaneously; 35.4% of them decide what to eat only on the day itself. The ratio of athletes who plan their meals a day in advance (26.5%) is similar to those who plan them 2–3 days ahead (29.2%). Greater awareness is less characteristic of our sample: the proportion of athletes who plan their menus 4–5 days or a week in advance is low (7.1% and 1.8%, respectively).

Ordinal regression was applied to analyze the effect of different variables (sport group, PENRAS, gender, age, previous consultation with a dietitian, and weekly endurance training volume) on meal planning habits (Table 3). The model was significant but had limited explanatory power. From the variables analyzed, the PENRAS seemed to be a significant positive predictor, suggesting that individuals with higher levels of nutrition knowledge are more likely to plan their meals in advance. Gender also had a significant impact on intentionality in planning meals ahead, indicating that women in the study group tended to plan their meals more in advance than men. Other variables (age, sport group, previous consultation with a dietitian, weekly endurance training volume) did not show significant effects (*p* > 0.05) on meal planning habits in our sample.

#### 3.4.2. Timing of Post-Workout Nutrition

The majority (77%) of respondents reported consuming their first meal within one hour after exercise, and only 4.4% waited for more than 2 h with their post-workout meal after finishing their training. The most common response (*n* = 45; 39.8%) in our sample was to consume the first meal within 60 min after exercising. 37.2% of respondents reported eating within 30 min post-exercise.

Regarding the consumption of energy-containing beverages, more than half of the respondents (53.1%) reported typically timing their first drink within 30 min after training. The proportion of energy-containing drink consumption within 60 min is 77.9% in our sample. Notably, 17.7% of respondents consume energy-containing drinks usually only more than two hours after exercise.

In the present study, post-exercise nutritional behaviors were investigated using two separate ordinal regression models: one focused on post-exercise meal timing, and the other on the consumption of energy-containing beverages during the post-workout period. The model evaluating the timing of post-workout meals had moderate explanatory power. Among the independent variables, knowledge score proved to be a significant predictor, indicating that athletes with higher levels of nutritional knowledge tend to eat sooner after exercise. Additionally, participants in the mixed sport group were more likely to eat earlier than triathletes.

The model examining the timing of energy-containing beverage consumption after exercise showed stronger explanatory power. In this case, nutritional knowledge was also a significant predictor. Athletes with higher knowledge levels were associated with shorter time intervals before consuming such beverages. Age also showed a significant negative association, suggesting that older athletes are more likely to consume energy-containing beverages earlier in the post-exercise period. Additionally, runners were significantly more likely to consume these beverages earlier than triathletes.

In addition to being familiar with the guidelines related to optimal post-workout nutrition, an athlete’s intention to lose weight may also influence the timing of meals. Based on Pearson’s Chi-squared test, respondents who want to lose weight are significantly more likely to intentionally postpone eating after exercise than those who do not have weight loss as a goal (*χ*^2^ (1) = 7.51; *p* = 0.006, *φ* = 0.258).

Additionally, according to Spearman’s rank correlation, a significant negative effect was observed between PENRAS and the behavior of intentionally avoiding nutrient intake within two hours after exercise (ρ = −0.23, *p* = 0.014). This result indicates that athletes with a higher knowledge of post-workout nutrition are less likely to deliberately delay their post-exercise meal and risk hindering the recovery process.

#### 3.4.3. Composition of the Post-Workout Meal

Participants’ post-exercise food choice preferences were analyzed by Wilcoxon signed-rank tests. Water consumption had the highest frequency within the two-hour period following exercise (Table 4). Consumption of the next main meal was significantly less frequent (*p* < 0.001) but still ranked at second place. A lower frequency of consumption was observed for fruits, protein shakes, sports drinks, snacks, coffee, fruit juice, and protein bars. The consumption of alcoholic beverages occurred with a significantly lower frequency rate than all other examined items (*p* < 0.001).

We employed an ordinal regression model to investigate the relationship between the consumption frequency of various food items and several predictors (Table 5). The dependent variable was the frequency of consumption in the two-hour post-exercise period (measured on a 5-point scale from “never” to “always”). Independent variables included PENRAS, gender, age, previous consultation with a dietitian, weekly endurance training volume, and sport group. The best fit and highest explanatory power among the models tested was observed by the model predicting protein shake consumption. According to our results, PENRAS significantly predicted the frequency of protein shake consumption in our sample, suggesting that athletes with higher levels of sports nutrition knowledge were more likely to consume protein shakes in the two-hour period after exercise. The ordinal regression model for fruit consumption after exercise also demonstrated a significant fit, with moderate explanatory power. In this case, the PENRAS showed a significant positive association with more frequent fruit consumption as well. Additionally, the variable sport group had a significant predictive power, as athletes in the mixed-sport group tended to consume fruit more frequently than triathletes. The model analyzing sports drink consumption was also statistically significant. Here too, PENRAS was a significant predictor, while no other variable had a significant effect on the frequency of sports drink consumption in the post-workout period.

## 4. Discussion

This study successfully filled a key gap in the literature by comprehensively examining the post-exercise nutrition knowledge and adherence to evidence-based recommendations of Hungarian endurance athletes, using the newly developed Post-Exercise Nutrition Recommendation Adherence Score (PENRAS). The significance of this assessment is underscored by the critical understanding that sports nutrition knowledge and the strategic application of dietary habits directly influence an athlete’s ability to achieve specific athletic results, particularly in high-volume endurance training and competition.

The analysis of nutrition-related goals clearly demonstrates the importance of sports performance among participants, as the majority (68.1%) indicated that they aimed to improve their sports performance. This goal significantly outweighs those related to body composition (e.g., weight loss or muscle gain) and alleviating digestive complaints. However, this focus exists alongside the adoption of potentially counterproductive dietary strategies: a notable proportion of athletes follow potentially restrictive diets, including low-carbohydrate (7.1%) approaches, which might hinder optimizing sports performance in endurance sports.

Consistent with results from validated questionnaires assessing general or sports-specific nutrition knowledge, the mean PENRAS in our cohort (*M* = 5.32 or 53.2%) falls within the range reported in the literature (between 40.2% and 70%) [9,24,27,28]. This suggests that while athletes’ knowledge of post-exercise recovery remains a critical area for improvement, the overall score is comparable to that of broader knowledge assessments. The relatively low standard deviation (*SD* = 1.52) and the maximum observed score of 8.5 points in our survey suggest that the overall level of knowledge is moderate, reinforcing the need for targeted educational intervention.

A large majority (84.1%) of participants correctly identified the importance of energy intake within the two-hour post-exercise window for optimal recovery. This result aligns well with the existing literature focusing on the sports nutrition knowledge of endurance athletes. A study analyzing sports nutrition knowledge in an international cohort of endurance athletes using the Comprehensive Evaluation of Athlete’s Knowledge Questionnaire (CEAC-Q) similarly reported a high success rate (84%) when assessing awareness about the importance of early carbohydrate consumption following glycogen-depleting exercise [27].

Knowledge regarding the quantitative amount of carbohydrate necessary for effective glycogen replenishment is alarmingly low, according to our results. Current guidelines recommend a high intake of carbohydrate (1–1.2 g·BW^−1^⋅h^−1^) during the initial four-hour recovery phase to ensure the fastest possible resynthesis of glycogen stores [11,12]. Over half of our participants (53.1%) reported not knowing the recommended carbohydrate amount for rapid recovery. Furthermore, the rate of providing the exact correct answer was alarmingly low at just 1.8%. However, when accepting the two options closest to the correct range, the proportion of participants giving an acceptable answer rose to 23%. This low level of knowledge is consistent with the literature. For instance, McLeman et al. found that only 1% of their endurance athlete sample correctly identified the recommended CHO intakes; the remaining respondents either selected ‘I don’t know’ (89%) or answered incorrectly [8]. Similarly, a survey of Australian triathletes by Doering and colleagues found that 43.1% responded “I don’t know” to the question on optimal carbohydrate content, with only 25.2% selecting the correct amount [7]. An international cohort assessed using the CEAC-Q reported that 29% of endurance athletes knew the exact amount recommended [27]. This consensus across different studies suggests that quantitative recovery nutrition is a relatively weak knowledge area among endurance athletes globally. Given the central role of glycogen in sustained endurance performance, this deficiency represents a major target for future sports nutrition education.

The difference observed in the success rates of the two factual knowledge items (high awareness regarding optimal timing vs. low knowledge of quantitative carbohydrate needs) is key to interpreting the overall PENRAS. This internal disparity highlights the difficulty in translating general awareness into specific, actionable nutritional strategies. The overall low average PENRAS, combined with literature showing low nutritional knowledge even among professional cohorts, suggests that athletes often lack the specific quantitative knowledge required for optimal, evidence-based performance fueling.

The high rate of participants (98.2%) correctly excluding high fat content when considering post-exercise meal composition shows awareness regarding the need to optimize for rapid nutrient absorption in the recovery phase. Consumption of mixed meals in the immediate post-exercise phase is known to significantly slow gastric emptying, thereby hindering the rapid delivery of carbohydrates essential for maximal muscle glycogen resynthesis [13]. On the other hand, our findings indicate a potential priority mismatch in macronutrient focus among our sample. The similar frequency with which athletes prioritized high protein (58.4%) and high carbohydrate (52.2%) content in their recovery meals suggests that athletes may be overly focused on muscle protein synthesis, potentially at the expense of adequately replenishing depleted glycogen stores. This imbalance was also reported by McLeman et al. According to their results, post-exercise carbohydrate intakes averaged close to the recommended minimum, but only 48% of participants actually achieved this target. Conversely, average protein intake post-exercise exceeded the minimum guidelines, with 75% of participants meeting or surpassing that lower threshold [8]. Doering et al. found that young triathletes met post-exercise recommendations, while professional athletes did not [7]. A further deficit was observed in our study in the low prioritization of easy digestibility (37.2%) and sodium replacement (23.9%).

The high proportion of athletes demonstrating a clear link between training load and nutrient intake indicates a functional awareness of macronutrient periodization. A significant 85% of participants indicated they increase their carbohydrate intake on intense training days. This dietary practice aligns perfectly with current scientific models of periodized nutrition [1]. Similarly, 55.8% of athletes reported consuming more protein when their training load is higher than usual. High protein intake during periods of increased stress is supported by evidence suggesting that higher protein availability may help enhance adaptive processes during high-volume training cycles [32].

The finding that triathletes achieved significantly higher PENRAS than runners and mixed-endurance athletes is noteworthy. This may be attributed to the necessity of managing short recovery windows inherent in triathlon training. Furthermore, the multi-sport nature and typically higher training complexity of triathlon may cultivate a higher sporting level and greater experience in nutritional strategy among participants. This environment may compel triathletes to more actively seek out specific, performance-oriented nutritional knowledge, particularly related to recovery, compared to single-sport athletes. However, our result stands in contrast to some previous research, and the literature remains inconsistent regarding knowledge differences across endurance disciplines. An evaluation of an international cohort of endurance athletes using the CEAC-Q did not support our findings, as triathletes in that study performed worse on the knowledge test than both cyclists and runners [27]. Another study utilizing the CEAC-Q found no significant differences in scores between cyclists, triathletes, and runners (*p* = 0.363) [33]. No significant difference between the knowledge levels of runners and triathletes was reported in a study using the ULTRA-Q tool either [28].

Besides the athlete’s primary sport, age was statistically associated with the PENRAS; older participants tended to reach lower scores in our sample. This finding aligns with previous research on knowledge of carbohydrate guidelines [27]. However, the literature is not entirely consistent on this point. For instance, Doering et al.’s work on triathletes found no statistically significant difference in knowledge between younger and master athletes [7].

Gender and education were not significantly associated with PENRAS in our sample. Trakman et al. found similar results in their review regarding gender differences: out of the fifteen studies reporting on male versus female knowledge scores, ten indicated no significant difference [19]. Knowledge levels did not differ between genders in a study using the ULTRA-Q questionnaire either [28]. In a study validating the PEAKS-NQ tool, Edmonds et al. reported females reaching slightly higher scores than males. According to their literature review, previous studies have come to similar results [34].

Previous consultation with a registered sports dietitian or nutritionist was a statistically significant predictor of the knowledge score in our sample. This finding strongly suggests a positive link with professional guidance and aligns with multiple research results showing enhanced nutritional literacy following consultation. For example, a study surveying collegiate athletes found that those who identified a sports dietitian as their primary nutrition information source demonstrated a greater understanding of nutrient periodization (47.12% correct answers vs. 32.85%) [30]. Furthermore, in an international cohort of endurance athletes, participants who consulted a registered sports nutritionist or dietitian achieved significantly higher CEAC-Q scores (58 ± 17% and 47 ± 20%, respectively, *p* < 0.001) [27]. However, the literature is not entirely consistent on the magnitude of this effect. In contrast, one study observed no significant difference in CEAC-Q scores between athletes who had worked with a dietitian and those who had not (56 ± 15% and 53 ± 16%; *p* = 0.419) [33]. It must be noted that this specific finding was drawn from a notably smaller sample size than the studies supporting this statement. Overall, the positive association in our and most other studies suggests that structured education by a nutrition professional is an effective approach. This conclusion is reinforced by a general review assessing the effectiveness of educational interventions, which reported that most structured programs lead to a significant improvement in nutrition knowledge [31].

The source of nutrition information was significantly associated with the PENRASs achieved by participants. The reliance on coaches and nutritional professionals was found to be significantly, positively associated with PENRASs. Conversely, seeking nutritional information from online sources showed a significant negative association. The most common response from the participants, their own experience was not found to be significantly associated with the score. Our findings are reinforced by other research: Sampson et al. found that athletes preferring credible information sources such as scientific journals or a registered sports nutritionist/dietitian, showed higher CEAC-Q scores than participants relying mostly on self-directed learning, coaches, other athletes, or friends and family [27]. Despite the demonstrated positive association of professional guidance, the likelihood of endurance athletes choosing nutritional professionals as their primary information source varies widely and often remains low across different studies. For example, Doering et al. found the most common response to the question about primary information source was “own previous knowledge” (17.3%), while accredited dietitians and nutritionists combined accounted for 6.3% of responses [7]. Similarly, McLeman et al.’s study of runners found that a dietitian or sports nutritionist was mentioned very rarely (1% and 2%, respectively), with the internet being the most frequently selected source [8]. However, the reported prevalence is highly dependent on the study population: Sampson et al. found a low rate of 14% choosing dietitians in an international endurance cohort [27] but noted a significantly higher prevalence (36%) in a more recent study, which might be attributed to the high rate of professional athletes in the sample [33]. This disparity suggests that while professional guidance is highly effective, barriers likely prevent most non-elite athletes from consistently seeking out or receiving expert advice.

Our analysis found that higher PENRASs were significantly associated with both better meal planning habits and the tendency to consume meals and energy-containing beverages earlier post-exercise. This suggests that better knowledge corresponds to better adherence to post-exercise meal timing recommendations. The PENRAS was significantly associated with more structured meal preparation, indicating that individuals with higher scores are more likely to plan their meals in advance. Interestingly, gender also had a significant association with this behavior (*B* = 0.84; *p* = 0.034), with women in the study group tending to plan their meals more in advance than men. Crucially, a higher PENRAS was also significantly associated with shorter time intervals before consuming the post-exercise meal and energy-containing beverages. This underscores the participants’ awareness of the critical timeframe for rapid glycogen replenishment, a finding reinforced by the negative correlation between PENRAS and the intentional delay of post-exercise meals.

The association between the PENRAS-score and specific post-exercise food choices was similarly significant. Higher knowledge levels were found to be significantly associated with more frequent consumption of protein shakes, fruits, and sports drinks in the two-hour post-exercise window. This suggests that greater sports nutrition knowledge aligns with the selection of foods known to facilitate the effective delivery of key nutrients necessary for rapid recovery.

Conversely, the relationship between theoretical knowledge and practice is not always direct. Sampson et al. did not find a clear link between CEAC-Q scores and actual nutrient intake during real-world competition, highlighting gaps in the practical application of knowledge, particularly for complex strategies like carbohydrate loading and in-competition intake [33]. The knowledge–practice gap might also appear in other behavioral components. For example, participants who listed weight loss as a nutritional goal were significantly more likely to intentionally postpone eating after exercise compared to those who did not. Many athletes believe that postponing their post-workout meals can support weight loss, and therefore adopt this habit, although this practice poses a significant risk of compromising glycogen resynthesis and thus recovery. Therefore, while our results support the association between knowledge and enhanced post-exercise timing adherence and key food choices, the translation to all dietary practices, especially those influenced by body composition concerns, requires further investigation.

The primary methodological limitation of our study is the relatively small sample size (*n*= 113), which restricts the generalizability of our findings and may have reduced the statistical power for detecting differences among certain subgroups. Furthermore, our study focused exclusively on a subpopulation of endurance athletes (runners, triathletes, and mixed-endurance athletes), which limits the generalizability of the findings across the broader endurance athlete community. Additionally, the recruitment method employed (specialist and snowball sampling) likely introduced a sampling bias towards individuals with higher educational attainment. Our sample had a high proportion of individuals with a university degree, meaning our conclusions apply primarily to a specific, highly literate and motivated subgroup of endurance athletes, rather than to amateur athletes in general. A further limitation relates to the resolution of data concerning participant experience and competitive level. Although our recruitment explicitly targeted amateur athletes training at least three times per week, we did not collect granular data on competitive history or total years of experience. This precludes a more nuanced analysis of how sporting level might modulate the observed adherence to sports nutrition guidelines. Moreover, our sample primarily consisted of non-elite, recreational athletes. While this provides valuable insight into the general athletic population, it is highly probable that competitive, professional athletes would have achieved higher PENRASs. Since a validated questionnaire on the specific topic of post-exercise nutrition was not yet available when the research was designed, we measured the knowledge level and adherence using self-developed questions. While grounded in current sports nutrition guidelines, the PENRAS is a newly developed index, and its external validity requires confirmation in larger, more diverse populations.

The current findings open up several pathways for future research aimed at assessing and optimizing the post-exercise nutritional practices of endurance athletes. Given the significant knowledge gaps identified, particularly concerning quantitative carbohydrate needs and the potential prioritization of protein over carbohydrates, future research should focus on the implementation and evaluation of targeted educational interventions. To provide more complete information on actual nutritional status and adherence to recommendations, future studies should combine the PENRAS with objective measures, such as detailed analysis of dietary records, body composition measurements, and the analysis of relevant biomarkers linked to recovery and training adaptation.

## 5. Conclusions

This study successfully addressed a gap in the literature by comprehensively assessing the post-exercise nutrition knowledge and adherence to evidence-based recommendations of Hungarian endurance athletes, using the newly developed Post-Exercise Nutrition Recommendation Adherence Score (PENRAS). The mean PENRAS (5.32 out of 10) indicates a basic understanding of recovery principles. However, substantial gaps remain in how athletes apply evidence-based carbohydrate recommendations. This knowledge gap is noted alongside the concerning prevalence of low-carbohydrate diets, which may contradict the athletes’ primary goal of maximizing performance. Our results underscore the critical role of information source quality: consultation with a dietitian was significantly associated with higher PENRASs, whereas reliance on online sources was negatively associated with the score. The study findings highlight a need for targeted educational interventions. These programs should prioritize specific education on the quantitative aspects of carbohydrate intake and actively redirect athletes towards reliable, evidence-based nutritional information to improve adherence, optimize glycogen replenishment, and ultimately support long-term performance outcomes. The growing availability and recognition of accredited sport dietitian programs in Hungary provide athletes with valuable opportunities to gain practical nutrition knowledge. Close collaboration with qualified dietitians can help them apply this expertise effectively in their training and recovery.

## Figures and Tables

**Table 1:** Participant characteristics by sport group (*n* = 112).

	Runners (*n* = 49)	Mixed Endurance Athletes (*n* = 36)	Triathletes (*n* = 27)
Gender			
Female	65.3%	50.0%	51.9%
Male	34.7%	50.0%	48.1%
Mean age (years) ± SD	38 ± 9	42 ± 9	40 ± 12
Highest level of education			
Vocational school	10.2%	8.3%	11.1%
Secondary school	10.2%	8.3%	18.5%
Higher education degree	79.6%	83.3%	70.4%
Ratio of participants who previously consulted a dietitian	49.0%	58.3%	63.0%

Values are presented as column percentages (%), unless otherwise indicated. Mean age is presented as the arithmetic mean.

**Table 2:** Responses for individual questions included in PENRAS.

Question	Answer Options	Frequency of Answers % (*n*)	Adherence Score
What is, in your opinion, the optimal timing for the first post-exercise meal when aiming for rapid recovery?	Timing does not matter	1.8% (2)	HIGH (84.1%)
	**Within 30 min**	**40.7% (46)**	
	**Within 60 min**	**35.4% (40)**	
	**Within 2 h**	**8% (9)**	
	Later than 2 h	0.9% (1)	
	I don’t know	13.3% (15)	
In your opinion, what is the optimal carbohydrate content of meals in the hours following endurance exercise if the goal is rapid recovery?	<0.4 g·BW^−1^⋅h^−1^	13.3% (15)	LOW (according to stricter criteria: 1.8%, according to less stringent criteria: 23%)
	0.4–0.8 g·BW^−1^⋅h^−1^	7.1% (8)	
	**0.8–1 g·BW^−1^** ⋅h^−1^ *	12.4% (14) *	
	**1–1.2 g·BW^−1^** **⋅h^−1^**	**1.8% (2)**	
	**1.2–1.6 g·BW^−1^** ⋅h^−1^ *	8.8% (10) *	
	>1.6 g·BW^−1^⋅h^−1^	3.5% (4)	
	I don’t know	53.1% (60)	
Do you consider the high energy content of food when planning your post-exercise meal?	**Yes**	**23.0% (26)**	LOW (23.0%)
	No	77.0% (87)	
Do you consider the high carbohydrate content of food when planning your post-exercise meal?	**Yes**	**52.2% (59)**	MODERATE (52.2%)
	No	47.8% (54)	
Do you consider the high sodium content of food when planning your post-exercise meal?	**Yes**	**23.9% (27)**	LOW (23.9%)
	No	76.1% (86)	
Do you consider the high protein content of food when planning your post-exercise meal?	**Yes**	**58.4% (66)**	MODERATE (58.4%)
	No	41.6% (47)	
Do you consider the high fat content of food when planning your post-exercise meal?	Yes	1.8% (2)	HIGH (98.2%)
	**No**	**98.2% (111)**	
Do you consider easy digestibility when planning your post-exercise meal?	**Yes**	**37.2% (42)**	MODERATE (37.2%)
	No	62.8% (71)	
Do you consume more carbohydrates on intense training days?	**Yes**	**85% (96)**	HIGH (85%)
	No	15% (17)	
Do you consume more protein on intense training days?	**Yes**	**55.8% (63)**	MODERATE (55.8%)
	No	44.2% (50)	

The bolding indicates statistical significance at *p* < 0.05. * Half points were given to the two options closest to the correct answer.

**Table 3:** A series of ordinal regressions explaining meal planning and nutrient timing behaviors. *B*-coefficients and *p*-values for each predictor across three ordinal regression models.

Predictor	Meal Planning ^1^ *B* (*p*)	Meal Timing ^2^ *B* (*p*)	Energy-Containing Beverage Timing ^3^ *B* (*p*)
PENRAS	0.28 **(0.038)**	−0.38 **(0.006)**	−0.75 **(<0.001)**
Gender (female)	0.84 **(0.034)**	0.14 (0.731)	0.40 (0.359)
Age	−0.01 (0.598)	−0.03 (0.139)	−0.05 **(0.023)**
Sport group: Mixed vs. Triathletes	0.47 (0.389)	−1.13 **(0.040)**	0.13 (0.818)
Sport group: Runners vs. Triathletes	0.81 (0.156)	−0.75 (0.189)	−1.65 **(0.012)**
Consulted dietitian	0.50 (0.190)	−0.19 (0.624)	0.29 (0.497)
Weekly endurance training volume	0.33 (0.303)	−0.01 (0.986)	−0.12 (0.731)

*B* = unstandardized regression coefficient. The bolding indicates statistical significance at *p* < 0.05. ^1^ Meal planning model: *χ*^2^ (7) = 19.66; *p* = 0.006; Nagelkerke *R*^2^ = 0.173; McFadden *R*^2^ = 0.066. ^2^ Meal timing model: *χ*^2^ (7) = 12.51; *p* = 0.085; Nagelkerke *R*^2^ = 0.117; McFadden *R*^2^ = 0.047. ^3^ Energy beverage timing model: *χ*^2^ (7) = 34.17; *p* < 0.001; Nagelkerke *R*^2^ = 0.294; McFadden *R*^2^ = 0.136.

**Table 4:** The frequency of consumption of various food items within two hours after exercise, as reported by participants on a five-point scale.

Food Item	Mean	Std. Deviation
Water	4.83	0.40
The next main meal	3.25	1.03
Fruit	2.97	1.11
Protein shake	2.28	1.39
Sports drink	2.27	1.17
Some type of snack	2.23	1.11
Coffee	2.17	1.14
Fruit juice	1.92	1.01
Protein bar	1.76	1.00
Alcoholic beverage	1.24	0.52

**Table 5:** Summary of ordinal regression results predicting post-exercise nutrition behaviors. *B*-coefficients and *p*-values for each predictor across three ordinal regression models.

Predictor	Protein Shake Consumption ^1^ *B* (*p*)	Fruit Consumption ^2^ *B* (*p*)	Sports Drink Consumption ^3^ *B* (*p*)
PENRAS	0.61 **(<0.001)**	0.46 **(0.001)**	0.40 **(0.003)**
Gender (female)	−0.58 (0.151)	−0.06 (0.871)	−0.28 (0.463)
Age	0.02 (0.332)	0.02 (0.295)	0.04 (0.086)
Sport group: Mixed vs. Triathletes	−0.36 (0.511)	1.25 **(0.020)**	−0.77 (0.145)
Sport group: Runners vs. Triathletes	0.28 (0.629)	0.95 (0.089)	−0.44 (0.426)
Consulted dietitian	0.53 (0.183)	0.11 (0.778)	0.17 (0.651)
Weekly endurance training volume	0.13 (0.702)	0.58 (0.073)	−0.35 (0.284)

*B* = unstandardized regression coefficient. The bolding indicates statistical significance at *p* < 0.05. ^1^ Protein shake consumption model: *χ*^2^ (7) = 27.59; *p* < 0.001; Nagelkerke *R*^2^ = 0.233; McFadden *R*^2^ = 0.089. ^2^ Fruit consumption model: *χ*^2^ (7) = 19.11; *p* = 0.008; Nagelkerke *R*^2^ = 0.166; McFadden *R*^2^ = 0.058. ^3^ Sports drink consumption model: *χ*^2^ (7) = 17.79; *p* = 0.013; Nagelkerke *R*^2^ = 0.156; McFadden *R*^2^ = 0.055.

## Data Availability

The data presented in this study are available on request from the corresponding author due to ethical reasons.

## Supplementary Materials

The following supporting information can be downloaded at: https://www.mdpi.com/article/10.3390/nu17223629/s1, Questionnaire S1: Analysis of Post-Exercise Nutrition Knowledge and Practices of Amateur Endurance Athletes.
