# Supplementary material for: Post-Exercise Nutrition Knowledge and Adherence to Recommendations Among Amateur Endurance Athletes
Source: Nutrients. 2025 Nov 20;17(22):3629. doi: 10.3390/nu17223629 (PMC12655176; doi:10.3390/nu17223629)
Supplement: Supplementary file 1 [file nutrients-17-03629-s001.zip › nutrients-3987779-Questionnaire S1.pdf]

Analysis of Post-Exercise Nutrition Knowledge and Practices of Amateur Endurance Athletes

I consent to the use of my data and responses from the survey 'Analysis of Post-Exercise Nutrition Knowledge and Practices of Amateur Endurance Athletes' for scientific analysis, presentations, and journal publication, provided they are kept strictly confidential.

- Yes
- No

<u>Sociodemographic data</u>

Gender:

- Male
- Female
- Prefer not to disclose

Age (years):

…………………………………

Highest Level of Education:

- Primary School
- Vocational School
- Secondary School
- Higher Education Degree
- Other: ………………………………………………………………

Height (cm):

…………………………………………..

Weight (kg):

………………………………………………

<u>Questions Related to Nutritional Habits</u>

Do you have any food allergies or intolerances?

- Yes
- No
- I am not aware of any

If yes, what allergy or intolerance is it?

……………………………………………………….

Do you follow any specific diet? (You may select multiple answers)

- No
- Ketogenic
- Low-carb
- Low-fat
- Gluten-free
- Dairy-free
- Vegetarian
- Vegan
- Other: ……………………………………………………………

Do you currently have any specific nutrition-related goals? (You may select multiple answers)

- No
- Weight loss
- Getting lean
- Muscle gain
- Improving sports performance
- Alleviate gastrointestinal complaints
- Other: ……………………………………………………………

How many meals do you consume on an average rest day?

…………………………………………..

How many meals do you consume on an average training day?

…………………………………………..

How far in advance do you generally plan your meals?

- Minimum 1 week in advance
- 4-6 days in advance
- 2-3 days in advance
- 1 day in advance
- I decide what to eat on the day itself

Does your nutrition differ based on the volume and intensity of your daily training?

- No
- Yes, higher intensity training reduces my appetite/food intake.
- Yes, when I train more, I increase my food intake

Which nutrients do you typically consume more of on days with more intense training loads? (You may select multiple answers)

- Overall, of all macronutrients
- Carbohydrate
- Protein
- Fat
- None
- Other: ………………………………………………………………

On days with a more intense training load, when do you typically eat more? (You may select multiple answers)

- During main meals
- I snack more between main meals
- Before training
- I consume sports nutrition products during training
- Immediately after training
- In the evening
- Never
- Other: ………………………………………………………………

What sources influence your nutritional habits? (1=Least important, 5=Most important)

- Own experience
- Advice from friends/acquaintances
- Magazines
- Trainer/coach
- Registered Dietitian or Nutritionist
- Online sources

Have you ever consulted with a dietitian or nutrition specialist?

- Yes
- No

If yes, please rate your experience on a scale of 1 to 5. (1=very negative, 5=very positive)

…………………………………………………….

Would you like to consult with a sports nutrition specialist in the future?

- Yes
- I don't find it necessary

If yes, what would be your primary goal for seeking expert support?

………………………………………………………

<u>Questions Related to Post-Exercise Nutrition</u>

How long after exercise do you usually consume your first meal or food item?

- Within 30 minutes
- Within 60 minutes
- Within 2 hours
- After more than 2 hours

How long after exercise do you typically consume a beverage containing calories (e.g., sports drink, recovery shake, fruit juice, etc.)?

- Within 30 minutes
- Within 60 minutes
- Within 2 hours
- After more than 2 hours

Does it ever happen that you consciously avoid consuming caloric food and drinks in the two hours following exercise?

- Yes
- No

If yes, for what purpose? (You may select multiple answers)

- I would like to lose weight
- I think it aids recovery processes more effectively
- I want to enhance fat metabolism
- Other: ………………………………………………………………

In your opinion, when is it advisable to consume the first post-exercise meal if the goal is rapid recovery?

- Timing does not matter at all
- Within 30 minutes
- Within 60 minutes
- Within 2 hours
- After more than 2 hours
- I don't know

Which aspects do you consider when planning your post-exercise meal? (You may select multiple answers)

- High energy content
- High vitamin content
- High sodium content
- High fat content
- High carbohydrate content
- High protein content
- Easy digestibility
- Other: ………………………………………………………………

In your opinion, what is the optimal carbohydrate content of meals in the hours following endurance exercise if the goal is rapid recovery?

- <0.4 g·BW^{-1}·h^{-1}
- 0.4-0.8 g·BW^{-1}·h^{-1}
- 0.8-1 g·BW^{-1}·h^{-1}
- 1-1.2 g·BW^{-1}·h^{-1}
- 1.2-1.6 g·BW^{-1}·h^{-1}
- >1.6 g·BW^{-1}·h^{-1}
- I don’t know

In your opinion, what is the optimal protein content of the post-endurance exercise meal if the goal is to promote recovery?

- <0.1 $g \cdot BW^{-1}$
- 0.1-0.25 $g \cdot BW^{-1}$
- 0.25-0.3 $g \cdot BW^{-1}$
- 0.3-0.5 $g \cdot BW^{-1}$
- 0.5-0.7 $g \cdot BW^{-1}$
- >0.7 $g \cdot BW^{-1}$
- I don't know

How often do you consume the following item in the 2 hours after training?

Item	1 (Never)	2 (Rarely)	3 (Sometimes)	4 (Often)	5 (Always)
Water	☐	☐	☐	☐	☐
Fruit Juice	☐	☐	☐	☐	☐
Sports Drink	☐	☐	☐	☐	☐
Alcoholic Beverage	☐	☐	☐	☐	☐
Coffee	☐	☐	☐	☐	☐
Protein Shake	☐	☐	☐	☐	☐
Protein Bar	☐	☐	☐	☐	☐
Fruit	☐	☐	☐	☐	☐
Some kind of snack	☐	☐	☐	☐	☐
The next main meal	☐	☐	☐	☐	☐

What do you consume most often after endurance-type exercise?

…………………………………………………………………………………………

Why do you choose that specific food/drink? (You may select multiple answers)

- It has practical packaging
- It tastes good/I crave it
- It is easily accessible
- Its carbohydrate content is adequate
- Its fat content is adequate
- Its protein content is adequate
- Its vitamin content is adequate
- Other, namely: ……………………………………………………

Questions Related to Dietary Supplement Consumption

Do you consume any dietary supplement(s)?

- Yes
- No

If yes, what dietary supplement(s) do you consume?

………………………………………………………………………………………..

What factors influenced your decision to select these products? (You may select multiple answers)

- Coach's recommendation
- Dietitian's/Nutritionist's recommendation
- Acquaintance's experience
- Opinions written in forums/Facebook groups
- Advertisement
- Pharmacist's recommendation
- Information found on websites
- Other:…………………………..

Questions Related to Fluid Consumption Habits

How much fluid do you consume on an average day?

- <1 liter
- 1-1.5 liters
- 1.5-2 liters
- 2-2.5 liters
- 2.5-3 liters
- >3 liters

Do you usually consume fluids during exercise?

- Yes
- No
- Varies

What kind of fluids do you typically consume after exercise? (You may select multiple answers)

- Water
- Sports drink (isotonic/hypotonic drink)
- Energy drink
- Protein shake
- Milk
- Fruit juice
- Other: …………………………………………………..

<u>Questions Related to Training</u>

Which of the following sports do you practice regularly? (You may select multiple answers)

- Triathlon
- Running
- Cycling
- Swimming
- Other: …………………………………………………..

On average, how many times per week do you engage in endurance-type exercise?

- <3 times
- 3-5 times
- 6-7 times
- >7 times

Average weekly training hours for the following sports activities:

	I do not practice it regularly	Max. 1 hour per week	1-2 hours per week	2-4 hours per week	4-6 hours per week	Min. 6 hours per week
Running						
Swimming						
Cycling						
Strength Training						
Other						

What are your short-term sports goals?

……………………………………………………………………………………………

Typical time of day for your workouts? (You may select multiple answers)

- Morning (before 9 am)
- Late morning (9 am–12 pm)
- Early afternoon (12 pm–4 pm)
- Late afternoon (4 pm–7 pm)
- Evening (after 7 pm)
